# Influence of cultivation substrate on antioxidant activities and triterpenoid profiles of the fruiting body of *Ganoderma lucidum*

**DOI:** 10.3389/fnut.2024.1329579

**Published:** 2024-02-07

**Authors:** Gelian Luo, Zhibin Pan, Zhibin Liu, Weiqing Cheng, Tingting Yu

**Affiliations:** ^1^Fujian Vocational College of Bioengineering, Fuzhou, China; ^2^College of Biological Science and Engineering, Fuzhou, China

**Keywords:** *Ganoderma lucidum*, cultivation substrate, triterpenoids, metabolomics, UHPLC-Q-Orbitrap-MS

## Abstract

**Introduction:**

The fruiting body of Ganoderma lucidum has been believed to possess a wide range of therapeutic effects. There are two main methods for artificial cultivation of G. lucidum to produce the fruiting body, namely wood log cultivation and substitute cultivation. The impact of cultivation substrates on the composition of bioactive compounds remains largely unexplored. This study aims to compare the antioxidant activities and triterpenoid profiles of the fruiting bodies of G. lucidum that cultivated through wood log cultivation (WGL) and substitute cultivation (SGL) methods.

**Methods:**

The antioxidant activities, including the DPPH radical scavenging, hydroxyl radical scavenging, superoxide radical scavenging, and total antioxidant activities, were assessed in both WGL and SGL samples. Furthermore, the UHPLC-Q-Orbitrap-MS technique was employed to compare their phytochemical profiles, with a specific emphasis on triterpenoid constituents.

**Results and discussion:**

It was found that WGL samples exhibited significantly higher total triterpenoid content, DPPH radical scavenging activity, and total antioxidant activity. Furthermore, an untargeted metabolomics approach employing UHPLC-Q-Orbitrap-MS tentatively identified a total of 96 triterpenoids. Distinguishingly different triterpenoid profiles between the two types of G. lucidum samples were revealed via the utilization of principal component analysis (PCA) and hierarchical cluster analysis (HCA). Specifically, 17 triterpenoids showed significant differences. Of these triterpenoids, 6 compounds, such as ganosporelactone B, ganoderol A, ganoderic acid A, ganoderic acid alpha, were significantly higher in SGL samples; 11 compounds, such as lucidenic acid A, lucidenic acid D1, lucidenic acid F, lucidenic acid G, lucidenic acid J, ganoderic acid E, and ganoderic acid O, were significantly higher in WGL samples. These findings expand our knowledge regarding the impact of cultivation substrate on the antioxidant activities and triterpenoid profiles of G. lucidum, and offer practical implications for its cultivation.

## 1 Introduction

*Ganoderma lucidum*, also known as Lingzhi or Reishi, is a fungal species belongs to the *Ganodermataceae* family (1). This fungus is a well-recognized medicinal mushroom, and holds a prominent position in traditional Chinese medicine for centuries due to its health benefits (2). The medicinal properties of *G. lucidum* has been extensively studied and documented in numerous literatures. Its consumption has been associated with a wide range of health benefits, including immunomodulatory effects (3), potent antioxidant activity (4), anti-inflammatory properties (5), and the ability to fortify liver health (6). Furthermore, it has been reported to positively impact cardiovascular health (7), promote sleep (8), and aid in managing various chronic conditions (7). These remarkable health benefits of *G. lucidum* have drawn the attention of scientific researchers, leading to numerous investigations aimed at uncovering the bioactive compounds responsible for such therapeutic effects.

*G. lucidum* possesses a diverse array of bioactive constituents that contribute to its therapeutic effects. This fungus is a rich source of triterpenes, polysaccharides, steroids, nucleotides, fatty acids, and other active secondary metabolites (2, 9, 10). Among these phytochemicals, the triterpenes, particularly the ganoderic acids and lucidenic acids, have been extensively studied and recognized for their pharmacological significance (11). Ganoderic acids, unique to this fungus, are classified into various classes, such as ganoderic acids A, B, C, D, and others (12). Ganoderic acids have garnered significant attention due to their potential anticancer effects. They have been shown to inhibit tumor growth, induce apoptosis, and suppress angiogenesis (12, 13). Their ability to inhibit tumor invasion and metastasis makes them promising candidates for novel anticancer therapies. Additionally, ganoderic acids exhibit hepatoprotective properties, protecting the liver from damage caused by various toxins and oxidative stress (12, 14). Studies have also revealed their anti-inflammatory effects, suggesting potential benefits in addressing different inflammatory disorders (15). Lucidenic acids, with a C27 lanostane skeleton, are the second largest group of triterpenoids identified in this fugus. They were also classified into lucidenic acid A, B, C, and others (16). Similar to ganoderic acids, various health benefits, such as antioxidant, anti-inflammatory, anti-cancer, of lucidenic acids have been documented (16). Apart from triterpenoids, the presence of steroids and polysaccharides in *G. lucidum* is believed to provide diverse health benefits (17, 18). These compounds, along with other phytochemicals, synergistically contribute to the versatile therapeutic potential of *G. lucidum*.

As *G. lucidum* is very scarce in nature, the wild collection of *G. lucidum* fruiting bodies is insufficient to meet the increasing demand, leading to a predominant reliance on artificial cultivation. Currently, two primary methods of artificial cultivation, namely wood log cultivation and substitute cultivation, are employed in the commercial production of *G. lucidum* fruiting bodies (19).

Wood log cultivation involves the utilization of hardwood logs, such as oak, maple, beech, and birch, to replicate the natural growth environment of *G. lucidum*, potentially resulting in high-quality products (19). On the other hand, substitute cultivation offers an alternative to the traditional wood log method by employing diverse substrates, including agricultural by-products or synthetic materials, as the growth medium. Compared to wood log cultivation, substitute cultivation offers several advantages, such as better control over growing conditions, increased yields, and the ability to recycle and reuse substrates (20). However, despite these benefits, the impact of cultivation substrates on the composition of bioactive compounds remains largely unexplored. Conducting a comprehensive comparison of the phytochemical profiles of *G. lucidum* grown using these two cultivation methods may provide valuable insights into optimizing its cultivation for the enhancement of its medicinal benefits.

Due to the comment believe that the fruiting bodies of *G. lucidum* cultivated through WGL may have better pharmacological effects than those from SGL, it is hypothesized that WGL samples have higher triterpenoid content and a distinct triterpenoid profile. In the present study, we utilized the UHPLC-Q-Orbitrap-MS technique to perform a comparative analysis of the phytochemical profiles, with a specific emphasis on triterpenoid constituents, within fruiting bodies of *G. lucidum* that cultivated through wood log cultivation and substitute cultivation methods. The primary objective of our study is to investigate and elucidate the impact of the cultivation substrate on the antioxidant activities and triterpenoid profiles of *G. lucidum*.

## 2 Materials and methods

### 2.1 Fungal materials

The dried fruiting bodies of *Ganoderma lucidum* (Leyss.ex Fr.) Karst used in this study were provided by GanoHerb Co. Ltd. (Fujian, China). For substitute cultivation, broadleaf tree sawdust (from oak, chestnut, olive, and peach trees), bran, corn flour, rice malt, gypsum powder, and other materials were used as the growth medium. After being bagged and sterilized, the fungal culture was introduced for cultivation until the fruiting bodies were fully developed. For wood log cultivation, trees suitable for *G. lucidum* growth were cut into specific lengths of logs. These logs were then bagged, sterilized, and inoculated with the fungal culture. As the mycelium grows through the logs, forming mycelial blocks, these blocks were buried in the soil for continued cultivation, leading to the growth of mature fruiting bodies. For each type of fruiting body, five individual samples were collected for chemical composition analysis.

### 2.2 Chemicals

The organic solvents utilized in the chromatographic analysis were purchased from CNW Technologies, Inc. (Düsseldorf, Germany) and were of UHPLC grade. 2-Chlorophenylalanine was purchased from HC Biotech (Shanghai, China) and was served as internal standard in LC–MS analysis. Ultrapure water was generated through the Millipore Alpha-Q water purification system (Millipore, Billerica, MA, United States). Test kits for the determination of total protein and polysaccharides content were purchased from Jiancheng Bioengineering Institute (Nanjing, China). All other chemicals were purchased from Huabo Chemical Reagents Co., Ltd. (Fuzhou, China) and were of analytical grade.

### 2.3 Total protein, polysaccharides, and triterpenoids content analysis

The total content of protein in the fruiting bodies of all *G. lucidum* samples was measured following the official method GB 5009.5-2016, National standards for determination of proteins in foods. Briefly, the dried fruiting body of all *G. lucidum* samples were pulverized and sieved through a 100-mesh screen. Subsequently, 100 mg of the obtained material was weighed and placed in flask, followed by the addition of a 5.0 mL sodium hydroxide solution (concentration 0.05 mol/L). Additionally, 20 mL of biuret reagent was introduced and vortexed for 15 min. Next, the mixture was allowed to settle at room temperature for 30 min. The resultant reaction mixture was centrifugated (4,000 rpm for 5 min). The supernatant was then analyzed using a spectrophotometer at a wavelength of 540 nm to determine absorbance. A calibration curve is constructed using bovine serum albumin solutions. The concentration of protein in the samples was then determined by comparing its absorbance with the calibration curve.

The total content of polysaccharides in the fruiting bodies of all *G. lucidum* samples was measured following the anthrone-sulfuric acid method as descripted by Chen et al. (21). Briefly, 2.0 g of power sample was placed in flask, followed by the addition of 60 mL water. After a one-hour period of sedimentation, the mixture was subjected to 4 h of reflux heating. After filtration with filter paper, the residue was re-extracted following the same extraction method. The combined filtrates were concentrated to remove water using a rotary evaporator. The residue was then dissolved in 5 mL of water, followed by the addition of 75 mL of ethanol. After a 12 h period of sedimentation at 4°C and centrifugation, the precipitate was dissolved in hot water and made up to a final volume of 50 mL. After centrifugation, 2 mL of the supernatant was taken and mixed with 6 mL of sulfuric acid-anthraquinone solution (0.1 g anthraquinone dissolved in 100 mL of sulfuric acid). After thorough mixing, the reaction mixture was allowed to stand for 15 min. The absorbance of the reaction solution was measured at a wavelength of 625 nm using a spectrophotometer. A calibration curve is constructed using glucose solutions. The concentration of polysaccharides in the samples was then determined by comparing its absorbance with the calibration curve.

The total content of triterpenoids in the fruiting bodies of all *G. lucidum* samples was measured following the colorimetric method descripted by Lu et al. (22). Briefly, 2.0 g of power sample was placed in flask, followed by the addition of 50 mL of ethanol. Ultrasound extraction was applied at a power of 140 W and a frequency of 42 kHz for a duration of 45 min. After filtration with filter paper, the residue was re-extracted following the same extraction method. The collected filtrates were combined. After centrifugation, 0.2 mL of the supernatant was taken and mixed with 0.2 mL of freshly prepared vanillin acetic acid solution (0.5 g of vanillin dissolved in 10 mL of acetic acid), along with 0.8 mL of concentrated perchloric acid. The mixture was thoroughly shaken, and then heated at 70°C in a water bath for 15 min. Following heating, the solution was allowed to cool to room temperature. Subsequently, 4 mL of ethyl acetate was added and mixed. The absorbance was measured at a wavelength of 546 nm. A calibration curve is constructed using oleanolic acid solutions. The concentration of triterpenoids in the samples was then determined by comparing its absorbance with the calibration curve.

### 2.4 Antioxidant activities analysis

#### 2.4.1 DPPH radical scavenging activity

The DPPH radical scavenging activity of the two types of sample was determined following the method reported by Mishra et al. (23), with some modifications. Briefly, 0.1 g of powered sample was placed in Eppendorf tube, and upon adding 1 mL of 80% methanol, the mixture was homogenized. Subsequently, ultrasound extraction was applied, using a power of 200 W for 30 min. After centrifugation (12,000 rpm, 10 min), the supernatant was taken and placed on ice until subsequent evaluation of DPPH radical, hydroxyl radical, superoxide radical scavenging activities, and total antioxidant activity. 150 μL sample extract and 150 μL DPPH solution (0.2 mmol/L in 80% methanol) were mixed and placed in darkness for 30 min. Then, the absorbance was measured at 517 nm with spectrophotometer. 150 μL sample extract and 150 μL 80% methanol mixture was used as control. 150 μL 80% methanol and 150 μL DPPH solution was used as blank control. The DPPH radical scavenging rate was calculated. The analysis was conducted in triplicate.

#### 2.4.2 Hydroxyl radical scavenging activity

The hydroxyl radical (·OH) scavenging activity was determined using the Fenton method (24), with some modifications. Briefly, the mixture of 50 μL sample extract, 50 μL salicylic acid–ethanol solution (9 mmol/L), 50 μL FeSO_4_ aqueous solution (9 mmol/L), 50 μL H_2_O_2_ (8.8 mmol/L), and 200 μL distilled water was incubated at 37°C for 20 min. Then, the absorbance was measured at 510 nm with spectrophotometer. As a blank control, 50 μL of 80% methanol was used to replace the sample extract. As a control, 50 μL distilled water was used to replace H_2_O_2_. The hydroxyl radical scavenging rate was calculated. The analysis was conducted in triplicate.

#### 2.4.3 Superoxide radical scavenging activity

The superoxide radical (·O_2_) scavenging activity was determined using a commercially available kit (G0129W, Grace Biotechnology, Suzhou, China), wherein 1,2,3-benzenetriol was utilized as a color developer. The absorbance of the reaction mixture was measured at 570 nm with spectrophotometer. The superoxide radical scavenging rate was calculated in accordance with the manufacturer’s instructions. The analysis was conducted in triplicate.

#### 2.4.4 Total antioxidant activity

The total antioxidant activity was determined by a commercially available kit (G0115W, Grace Biotechnology, Suzhou, China), using Ferric-reducing antioxidant power (FRAP) method. The absorbance of the reaction mixture was measured at 590 nm with spectrophotometer. The analysis was conducted in triplicate. Additionally, a calibration curve was established for a series of trolox solutions (0, 2.5, 5, 10, 15, and 20 μmol/mL in 80% methanol) to facilitate the computation of the trolox equivalent for both samples.

### 2.5 Triterpenoid profile analysis

#### 2.5.1 Sample extraction

The dry SGL and WGL samples were subjected to grind to obtain a fine powder. 0.5 g of the powder was then subjected to extraction using 2.5 mL of a solution composed of 25% methanol in water, which also contained an internal standard of 2-chlorophenylalanine at a concentration of 1 μg/mL. The extraction process was carried out in an ultrasonic ice-water bath for a duration of 60 min. Following extraction, the resulting mixture was passed through a 0.22 μm filter and then centrifuged (12,000 rpm, 20 min, 4°C). From the resulting solution, an aliquot of 300 μL supernatant was collected for further analysis in the untargeted metabolomic investigation. Additionally, a quality control (QC) sample was prepared by mixing equal aliquots of supernatants from all the samples. This QC sample served as a reference for assessing the reproducibility and reliability of the analysis.

#### 2.5.2 Ultra-high performance liquid chromatography analysis

Chromatographic analysis of the extracts of SGL and WGL samples was performed on a Vanquish UPLC system (Thermo Fisher Scientific, Bremen, Germany) with an Acquity UPLC BEH C18 column (2.1 mm × 100 mm, 1.7 μm; Waters, Milford, MA). The mobile phases consisted of 0.1% (v/v) formic acid in water (solvent A) and 0.1% (v/v) formic acid in acetonitrile (solvent B) with a flow rate of 500 μL/min. The gradient program was: 85% solvent A (0 min) → 25% solvent A (11 min) → 2% solvent A (12 min) → 2% solvent A (14 min) → 85% solvent A (14.1 min) → 85% solvent A (16 min). The injection volume was set at 5 μL. The QC sample was injected once at the beginning, in the middle, and at the end of the run to obtain three data to monitor the stability of instrument.

#### 2.5.3 Mass spectrometry analysis

Following the chromatographic separation, high-resolution MS data were recorded using a Q Exactive Focus Orbitrap mass spectrometer (Thermo Fisher Scientific, Bremen, Germany). The instrument was equipped with a heated-electrospray ionization II (HESI-II) source, operating in both positive and negative ESI modes. Specifically, the following source parameters were employed: a spray voltage of 4.0 kV for both positive and negative modes, with nitrogen sheath gas flowing at a rate of 45 Arb and nitrogen auxiliary gas flowing at 15 Arb. The capillary temperature was set at 400°C. For MS scanning, the acquisition scan range was set between 100 and 1,500 m/z, with a full MS scan resolution of 70,000 full width at half maximum (FWHM) and data-dependent MS/MS scan resolution of 17,500 FWHM. Prior to the analysis, an external calibration was meticulously conducted to ensure accurate mass measurements, adhering to the manufacturer’s guidelines. The acquired data was subsequently processed using Xcalibur 4.0 software (Thermo Fisher Scientific, Bremen, Germany). For elemental composition prediction of the detected components, the following settings were applied: elements considered were carbon (C) up to 80 atoms, hydrogen (H) up to 130 atoms, and oxygen (O) up to 60 atoms. The mass tolerance was set to be less than 5 ppm, and compounds with a ring double-bond equivalent (RDBeq) value equal to or greater than 7 were taken into consideration.

#### 2.5.4 Data processing and multivariate statistical analysis

Raw data of all samples that acquired from the UHPLC-Q-Orbitrap-MS was first converted to the mzXML format by msConvert software (version 3, ProteoWizard) and then processed by using XCMS package in R software (Version 3.6.1, R Core Team, New Zealand) for peak extraction, peak alignment and peak integration. In order to comprehensively analysis the chemical composition of samples, data processing was performed in both positive and negative ionization modes. In order to dig useful information from the acquired data, multivariate statistical analyses, including the principal component analysis (PCA) and hierarchical cluster analysis (HCA), were performed by using R software based on the output data from XCMS. For identification of the metabolites in the samples, the detected ion features from UHPLC-Q-Orbitrap-MS were qualitatively analyzed based on the in-house metabolite database (Shanghai Biotree biotech Co., Ltd.) and the public database, such as HMDB, METLIN, *M/Z*cloud. Furthermore, in order to filter the differential metabolites between the two types of samples, volcano plot analysis was performed.

## 3 Results and discussion

### 3.1 Total protein, polysaccharides, and triterpenoids content comparison

**Figure 1:**
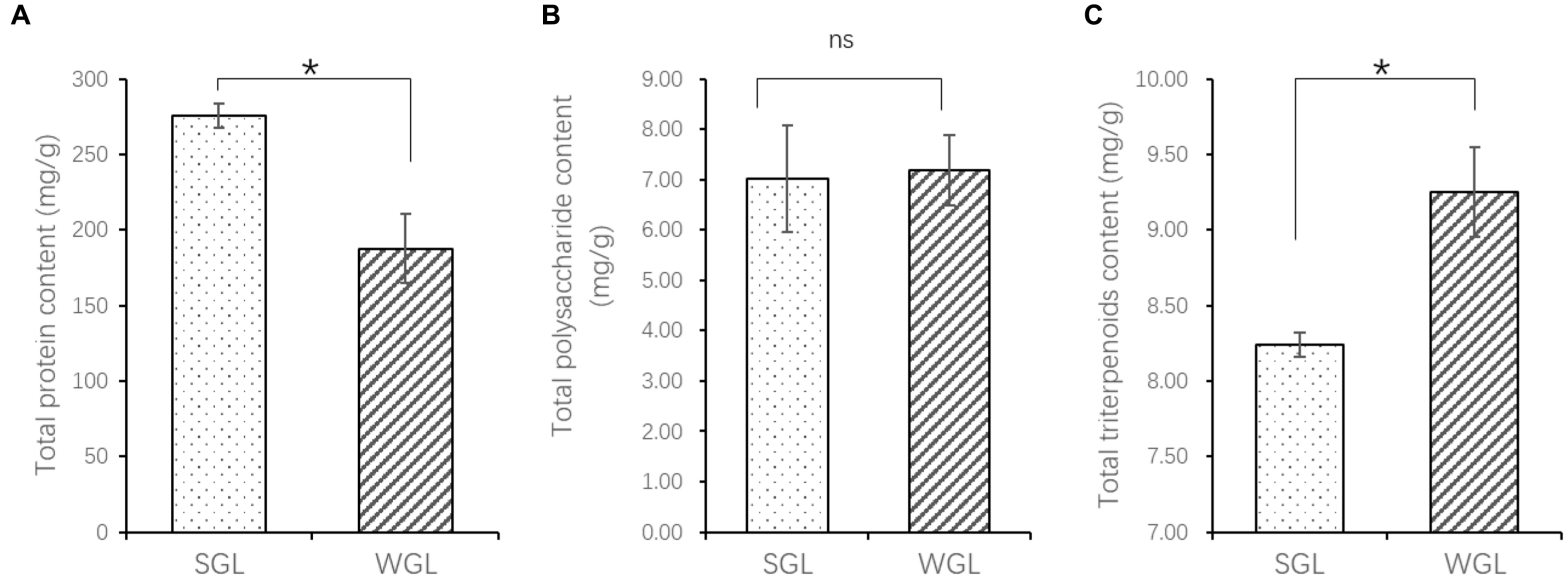
The content of total protein **(A)**, polysaccharides **(B)**, and triterpenoids **(C)**. The symbol “*” indicates the *p* < 0.05; “ns” indicates no significant difference.

The contents of protein and triterpenoids exhibited significant differences between the two types of samples, as shown in Figure 1. Specifically, SGL samples showed a significantly higher protein content (*p* = 0.012), while WGL samples showed a significantly higher total triterpenoid content (*p* = 0.028). Conversely, no statistically significant difference was observed in polysaccharide content between the two sample types (*p* > 0.05).

### 3.2 Antioxidant activities comparison

**Figure 2:**
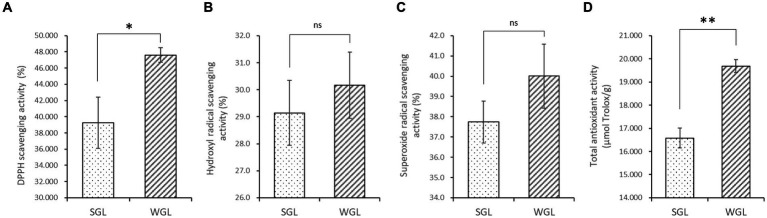
The antioxidant activities, including the DPPH radical scavenging **(A)**, hydroxyl radical scavenging **(B)**, superoxide radical scavenging **(C)**, and total antioxidant **(D)** activities. The symbol “*” indicates the *p* < 0.05; “**” indicates the *p* < 0.01; “ns” indicates no significant difference.

The antioxidant activities, including the DPPH radical scavenging, hydroxyl radical scavenging, superoxide radical scavenging, and total antioxidant activities, were assessed in both WGL and SGL samples. As shown in Figure 2, WGL samples exhibited significantly higher DPPH radical scavenging activity (*p* = 0.0036) and total antioxidant activity (*p* = 0.001) in comparison to SGL samples. It is well-established in the literature that triterpenoids and polysaccharides in *G. lucidum* exert potent free radical scavenging activities (25). The higher triterpenoid content detected in the WGL samples explained the superior DPPH radical scavenging and total antioxidant activities in comparison to the SGL samples.

### 3.3 General phytochemical profiles comparison

**Figure 3:**
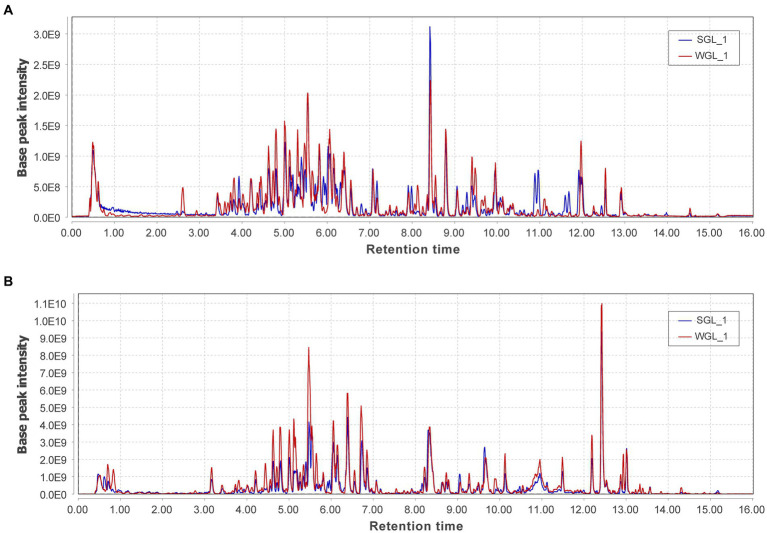
The representative base peak chromatograms of the two types of P *G. lucidum* fruiting bodies obtained from negative **(A)** and positive **(B)** ionization modes.

Untargeted metabolomic methodologies, involving high-resolution mass spectrometry and chemometric tools, have been effectively employed to analyze the chemical composition of diverse botanical specimens, such as tea leaves (26) and rhizomes of *Polygonatum sibiricum* (27). In this study, an untargeted metabolomic approach utilizing UHPLC-Q-Orbitrap-MS was adopted to comprehensively profile the chemical constituents of ten samples of *G. lucidum* fruiting bodies. The representative base peak chromatograms in both negative and positive ionization modes, revealed abundant metabolite information (Figure 3). The positive ionization mode exhibited more peaks and higher intensities compared to the negative ionization mode. Comparison between the two types of samples displayed comparable metabolic profiles in both ionization modes. A more detailed analysis of the chromatograms revealed that although many peaks overlapped, they exhibited variations in intensities.

**Figure 4:**
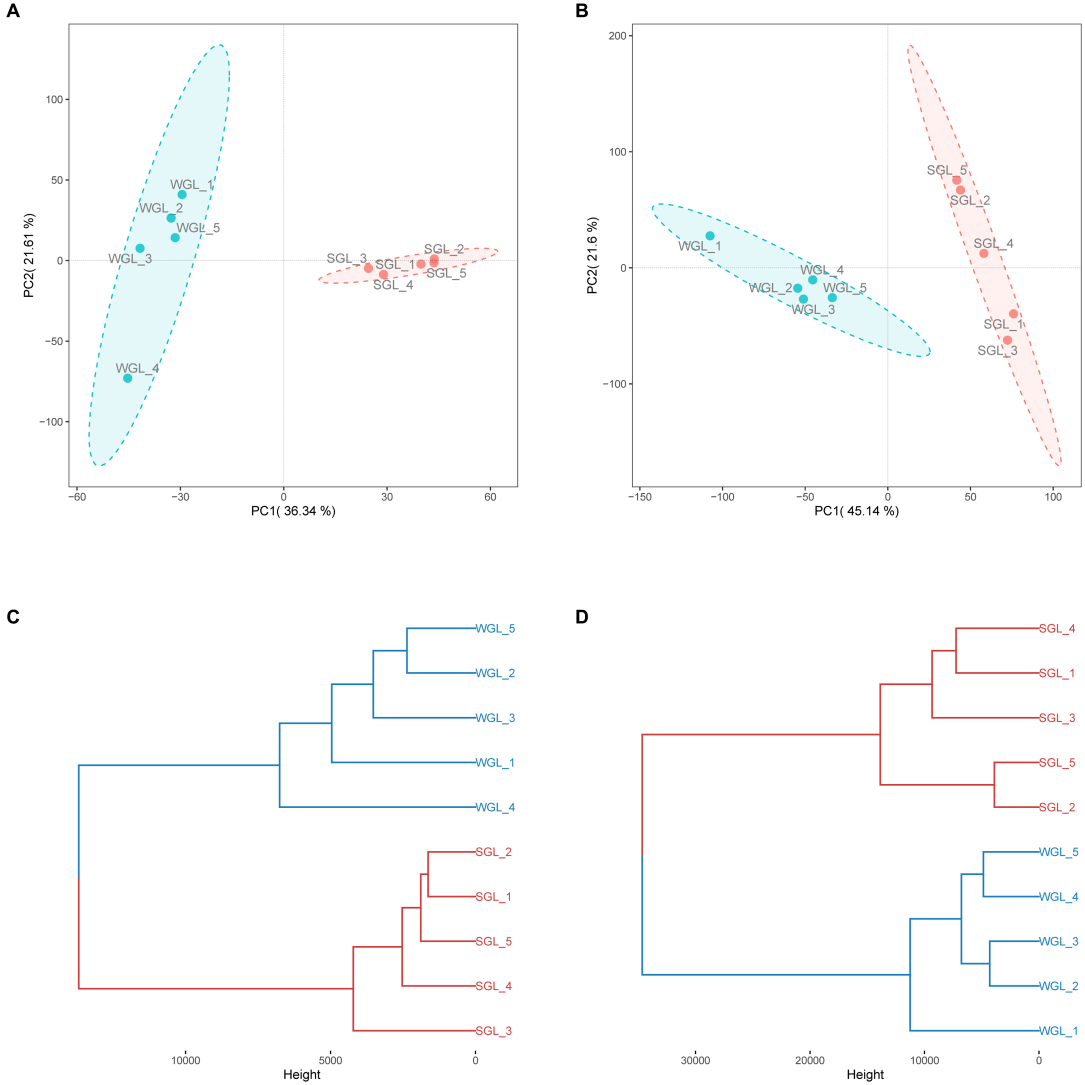
General metabolite profiles comparison. PCA score plots of the metabolites obtained from negative **(A)** and positive **(B)** ionization modes, and HCA dendrograms of the metabolites obtained from negative **(C)** and positive **(D)** ionization modes.

The software mzMine was further employed for chromatographic peak detection, alignment, filtration, and extraction, yielding two data matrices with 4,147 and 9,449 ion features in negative and positive ionization modes, respectively. Subsequently, to further elucidate the overall metabolic profiles of the two *G. lucidum* fruiting body sample types, chemometric tools, notably PCAHCA, were implemented. The ion features obtained from UHPLC-Q-Orbitrap-MS analyses in both ionization modes were input into R software for PCA and HCA assessments. The unsupervised PCA demonstrated the distribution of all samples in reduced dimensions. As depicted in Figures 4A,B, PCA score plots for positive and negative ionization modes exhibited distinct groupings, aligned with the sample types. Subsequently, HCA was conducted to classify samples with similar metabolic profiles, as illustrated in Figures 4C,D. Notably, a clear grouping pattern consistent with the sample types emerged in both dendrograms representing positive and negative ionization modes.

Collectively, the chromatographic profiles indicated a likeness in the general metabolic composition of *G. lucidum* fruiting bodies cultivated on wood logs and substitute media. However, PCA and HCA unveiled distinctive differences in their metabolic profiles, implying variations in specific metabolites. Detailed insights into the metabolites, particularly triterpenoids, are presented and discussed further below.

### 3.4 Triterpenoid profiles comparison

A total of 176 and 527 phytochemical compounds were tentatively identified in the negative and positive modes of the mass spectra, respectively. These phytochemicals were categorized into aliphatic acyls, alkaloids, amino acid derivatives, flavonoids, fatty acids, phenolic acids, and terpenoids. A noteworthy subset within terpenoids, referred to as triterpenoids, has garnered significant attention due to their robust research focus and pharmacological relevance within *G. lucidum*. In the present study, a total of 96 triterpenoids, including ganoderic acids, ganoderiols, ganolucidic acids, ganosporelactones, lucidenic acids, and various other structurally diverse compounds, were putatively identified. Considering their pharmacological significance, the following study mainly focus on the comparison of the triterpenoid profiles within the *G. lucidum* fruiting bodies. The detailed information of the detected and identified triterpenoids, including their compound names, composite score, molecular formula, class, accurate mass, retention time, and the integrated peak areas in all samples was provided in Supplementary Table S1. It is noteworthy that samples from WGL demonstrated higher total triterpenoids than those from SGL, with an approximately 1.2-fold increase in the total peak areas of all 98 triterpenoids identified. This result is in line with the determination of the total triterpenoids using colorimetric method, as shown in Figure 1. Next, the general triterpenoid profiles and some individual distinctive triterpenoid compounds between SGL and WGL samples are further compared.

#### 3.4.1 General triterpenoid profile comparison

**Figure 5:**
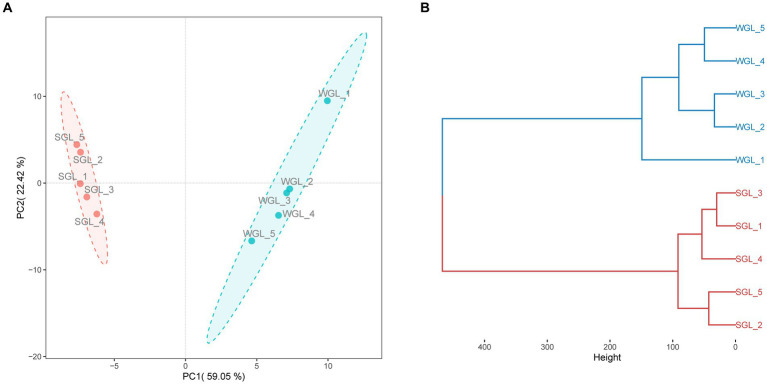
General triterpenoid profiles comparison. PCA score plots of triterpenoids **(A)**, and HCA dendrograms of triterpenoids **(B)**.

PCA was conducted to assess the distribution of triterpenoid constituents within the two distinct sample categories in a dimensionality-reduced space. As illustrated in Figure 5A, the PCA score plots exhibit the segregation of all samples into two distinctive clusters, concordant with their respective sample types. Similarly, a clear grouping pattern, consistent with the sample types, was observed in HCA (Figure 5B). Therefore, the results derived from both PCA and HCA collectively indicated that the general triterpenoid profiles present in the *G. lucidum* fruiting body, cultivated via wood log and substitute methodologies, exhibited substantial dissimilarity.

**Figure 6:**
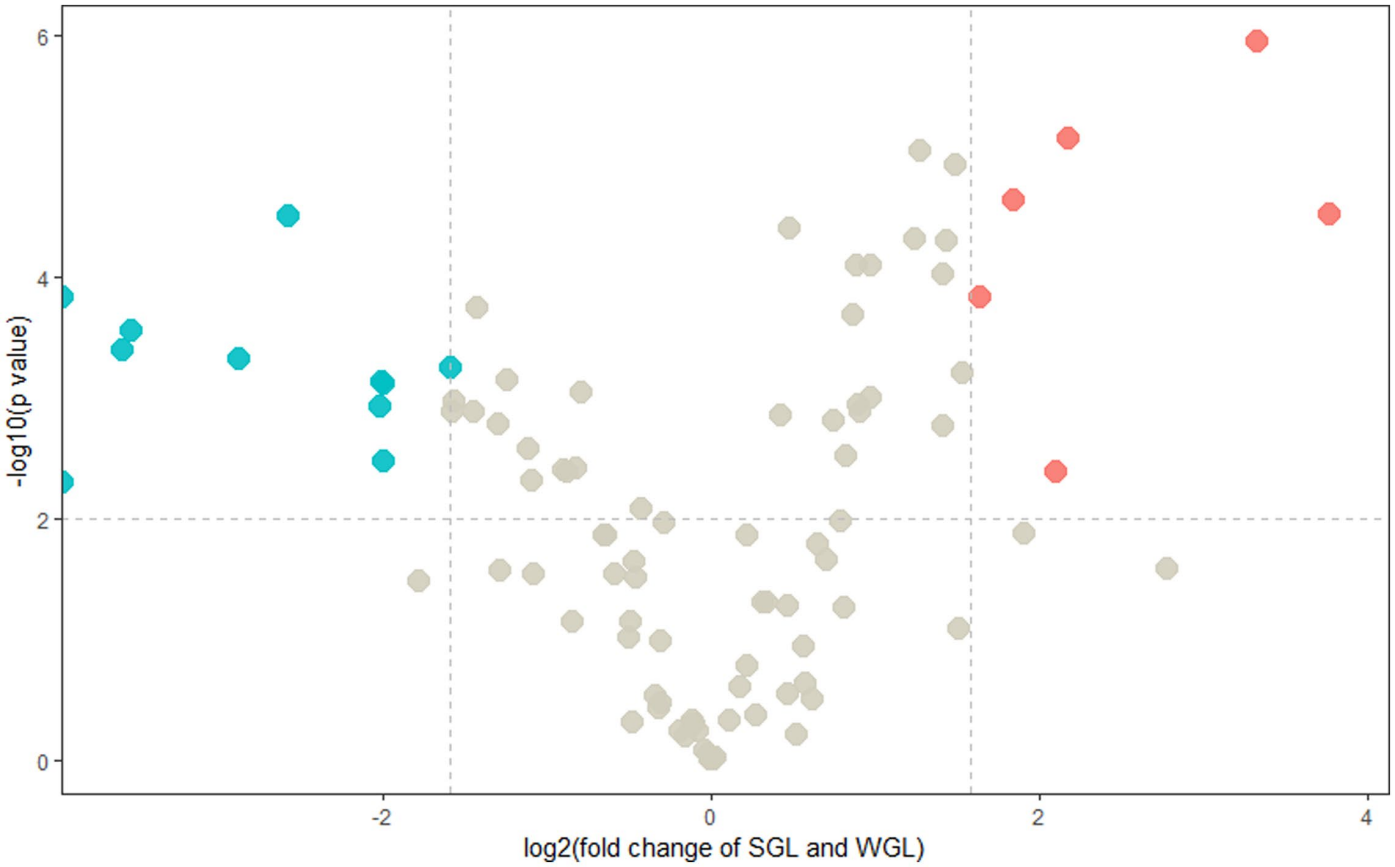
Volcano plots of the identified triterpenoids of the SGL and WGL samples.

In order to discriminate distinctive triterpenoids, a volcano plot was employed to compare individual triterpenoids across the two types of samples. In the volcano plot, the vertical axis represented the negative base 10 logarithm of the *p*-value, while the horizontal axis represented the logarithm base-2 of the fold change between SGL samples and WGL samples. Each plotted point in the volcano plot corresponded to a triterpenoid compound identified. Triterpenoid compounds that exhibited a fold change ≥3.0 or ≤ 0.33, accompanied by a *p*-value of <0.01, were categorized as differentially changed compounds. Accordingly, such compounds were color-coded as red, which were indicative of significantly elevated in SGL samples (fold change ≥3.0, and *p*-value < 0.01), or color-coded as blue, which were indicative of significantly elevated in WGL samples (fold change ≤0.33, and *p*-value < 0.01). The outcomes of this analysis are presented in Figure 6. It can be seen that six triterpenoids exhibited noteworthy elevation in SGL samples, while 11 triterpenoids exhibited noteworthy elevation in WGL samples. Detained information of these 17 differentially elevated triterpenoids is listed in Table 1.

**Table 1:** The differentially elevated triterpenoids.

ID	Tentative identification	Formula	Measured m/z	RT (min)	Δ m/z (ppm)	MS2	Average peak area	
							SGL	WGL
1	Ganosporelactone B	C_30_H_42_O_7_	515.2996	5.4	0.82	497.288;479.281;461.264;139.076;115.039	6.72E+09	4.96E+08
2	Ganoderic acid A	C_30_H_44_O_7_	517.3162	4.56	0.42	499.304;481.297;463.282;139.075;517.309	1.04E+09	2.43E+08
3	Ganoderol A	C_30_H_46_O_2_	439.357	10.65	0.09	439.359;421.344;81.07;109.101;69.07	2.21E+08	2.22E+07
4	Ganoderic acid alpha	C_32_H_46_O_9_	575.32	7.57	1.68	497.289;92.666;461.264;479.28;69.033	5.88E+06	1.31E+06
5	Polyporusterone F	C_28_H_46_O_5_	463.3421	2.92	2.41	463.284;445.271;95.084;269.188;113.096	2.14E+08	6.00E+07
6	Ixocarpanolide	C_28_H_40_O_6_	473.2903	5.75	0.55	473.286;455.28;81.07;92.666;55.054	1.21E+08	3.89E+07
7	Lucidenic acid F	C_27_H_36_O_6_	457.2589	4.84	0.32	457.303;439.291;83.086;81.07;71.049	9.16E+07	6.72E+08
8	Ganoderic acid E	C_30_H_40_O_7_	513.2837	3.43	0.58	495.275;513.288;69.033;477.267;139.076	1.94E+07	7.80E+07
9	Lucidenic acid G	C_27_H_40_O_7_	477.2852	2.35	0.38	70.065;423.253;441.258;477.283;293.151	6.39E+06	7.42E+07
10	Lucidenic acid J	C_27_H_38_O_8_	491.2645	2.98	0.94	473.253;491.256;437.233;99.044;419.216	1.49E+07	6.02E+07
11	Lucidenic acid D1	C_27_H_34_O_7_	471.2376	5.26	0.90	471.23;435.211;417.207;389.207;407.224	0	4.98E+07
12	Lucidenic acid A	C_27_H_38_O_6_	459.2733	2.35	1.52	459.27;441.258;70.065;423.25;121.064	5.95E+06	2.37E+07
14	Ganoderic acid O	C_30_H_40_O_8_	529.2795	6.21	0.86	511.271;139.076;69.033;529.276;483.276	2.44E+07	7.36E+07
13	11-Anhydro-16-oxoalisol A	C_30_H_46_O_5_	451.32	10.5	0.02	451.318;452.337;433.306;124.039;105.07	3.27E+07	1.32E+08
15	24-epi-brassinolide	C_28_H_48_O_6_	479.3384	9.29	0.74	479.343;92.685;443.316;461.329;59.714	1.34E+06	8.04E+06
16	Pomolic acid	C_30_H_48_O_4_	473.3625	6.62	1.06	473.367;111.08;81.07;161.131;455.346	0	2.26E+06
17	Verazine	C_27_H_43_NO	398.3418	14.07	0.61	398.345;61.248;69.07;59.775;57.633	9.64E+04	1.16E+06

#### 3.4.2 Differentiating triterpenoids

Six triterpenoids, namely ganosporelactone B, ganoderol A, ganoderic acid A, ganoderic acid alpha, polyporusterone F, and ixocarpanolide, were found to have significant higher abundance in SGL samples. On the other hand, 11 triterpenoids, namely lucidenic acid A, lucidenic acid D1, lucidenic acid F, lucidenic acid G, lucidenic acid J, ganoderic acid E, ganoderic acid O, 11-anhydro-16-oxoalisol A, 24-epi-brassinolide, pomolic acid, and verazine, were found to have significant higher abundance in WGL samples.

**Figure 7:**
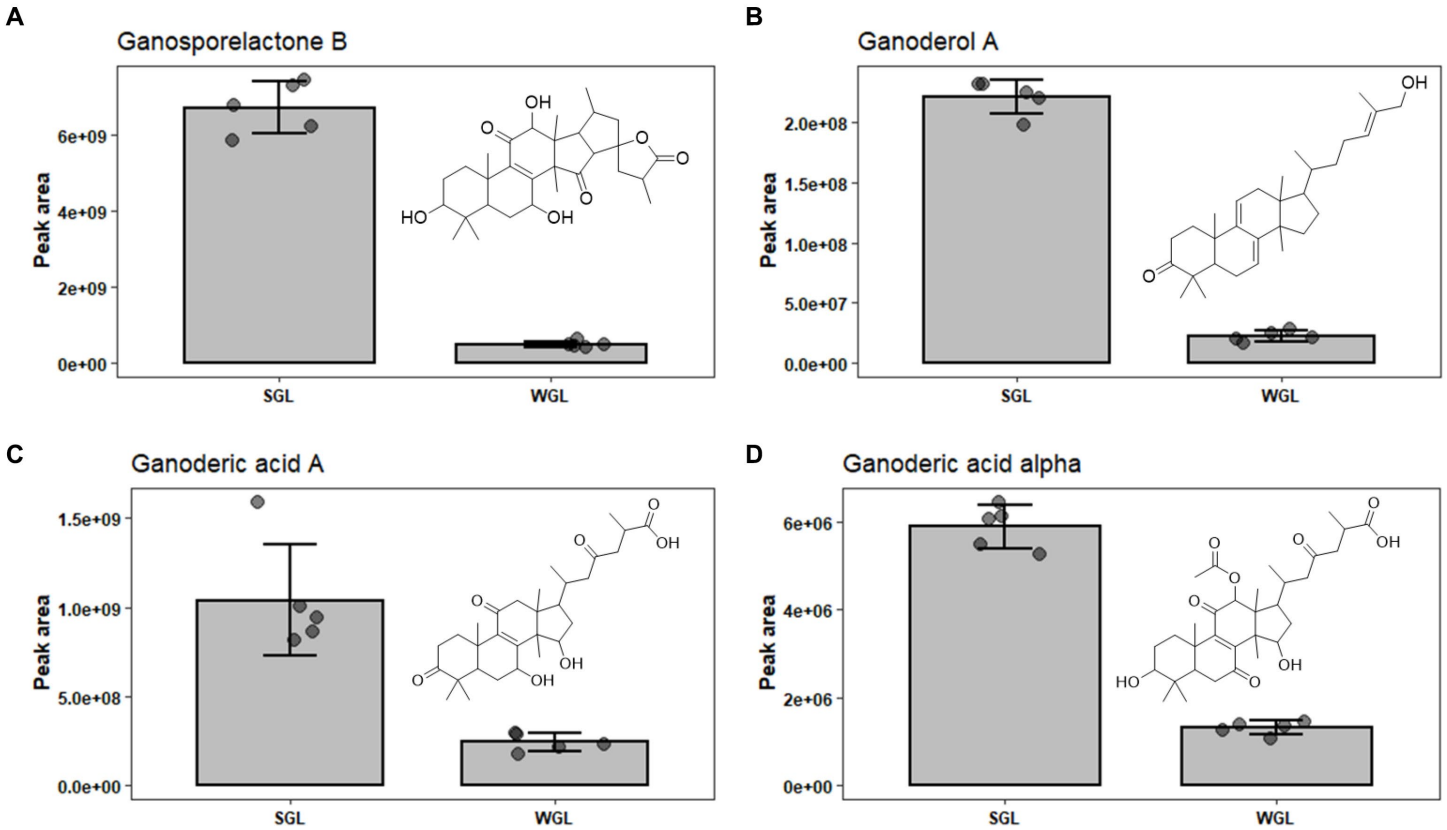
The comparison of the content of ganosporelactone B **(A)**, ganoderol A **(B)**, ganoderic acid A **(C)**, and ganoderic acid alpha **(D)** in SGL and WGL samples.

Ganosporelactone A and B have been isolated and identified in *G. lucidum* spores. These two triterpenoid lactone compounds could potentially originate from lanostane skeleton through the construction of C16 and C23 bond (28). As shown in Figure 7A, a particularly notable finding was the substantial difference in ganosporelactone B content between the two cultivation methods. The SGL samples exhibited a remarkable 13.5-fold increase in ganosporelactone B content compared to the WGL samples. This suggests that the substitute cultivation method has a significant impact on the biosynthesis or accumulation of ganosporelactone B. This could be attributed to differences in nutrient availability, or genetic expression induced by the substitute cultivation process. Ganosporelactone B is known for its potential bioactivity and health benefits, including anti-inflammatory and antioxidant properties (29). The higher content of this compound in SGL samples could enhance the potential therapeutic effects of *G. lucidum* products cultivated through this method. In contrast to ganosporelactone B, the study found comparable levels of ganosporelactone A across all tested samples, irrespective of the cultivation method used. This suggests that the biosynthesis or accumulation of ganosporelactone A might be less influenced by the cultivation method and more conserved in *G. lucidum*.

Ganoderol compounds constitute a cluster of triterpenoids prevalent in *Ganoderma lucidum*. These compounds are characterized by their tetracyclic triterpenoid structure, which includes multiple rings and functional groups. There are several types of ganoderol compounds, including ganoderol A, B, C, D, and F (30). Among these five ganoderols, ganoderol A is one of the prominent ganoderol compounds found in *G. lucidum*. Ganoderol A is classified as a lanostane-type triterpenoid, with a tetracyclic structure consisting of four fused rings. It has been a subject of research due to its potential health-promoting properties, including antioxidant and anti-inflammatory effects (31). Our study revealed substantial differences in the content of ganoderol A between SGL and WGL samples, with SGL samples containing a notable 10-fold increase in ganoderol A compared to WGL samples, as shown in Figure 7B. This finding points to a clear impact of the cultivation method on the production of ganoderol A. Additionally, our study identified the presence of ganoderol B, D, and F in both cultivation methods, with their content being 1.02, 0.66, and 1.87-fold of those in WGL, respectively. The observed increase in the content of ganoderol A and F in SGL samples suggests that the substitute cultivation method may provide a more conducive environment for the biosynthesis or accumulation of these two triterpenoids. The potential functional implications of these compounds, which have been linked to diverse health-promoting effects such as immunomodulation and cardiovascular protection, further highlight the importance of understanding the cultivation-driven variability in their content.

**Figure 8:**
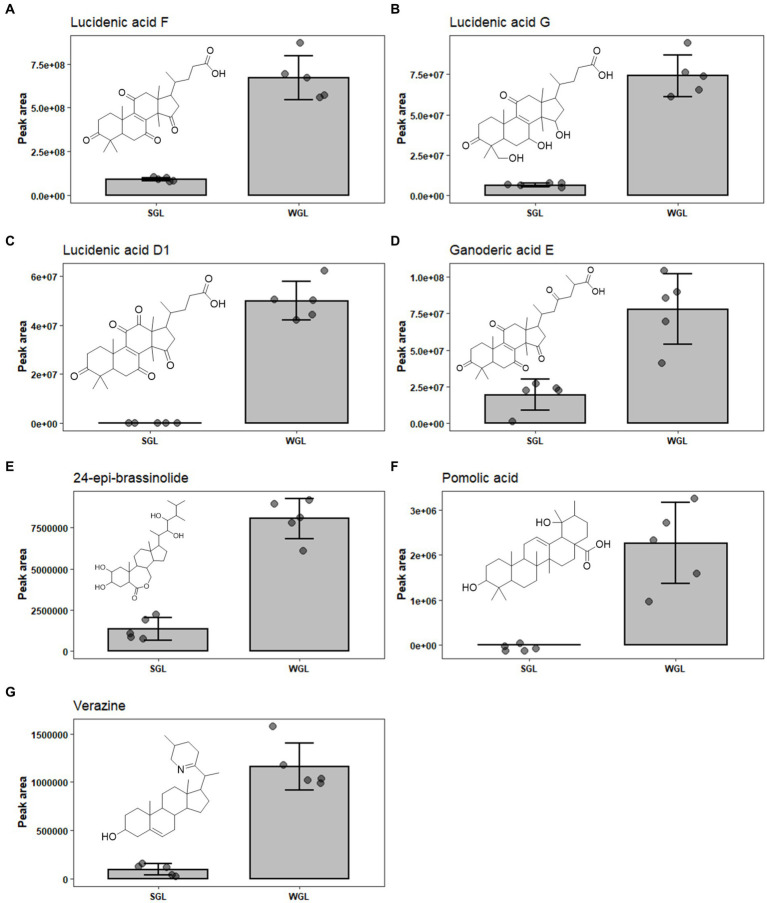
The comparison of the content of lucidenic acid A **(A)**, lucidenic acid D1 **(B)**, lucidenic acid F **(C)**, lucidenic acid G **(D)**, lucidenic acid J **(E)**, ganoderic acid E **(F)**, and ganoderic acid O **(G)** in SGL and WGL samples.

Upon analyzing the results, it is evident that both cultivation methods resulted in the identification of an array of lucidenic and ganoderic acids, which are known to be the primary triterpenoids in fruiting body of *G. lucidum.* Lucidenic acids, with a characteristic C27 lanostane skeleton and a side chain of carboxyl group, are the second major group of triterpenoids found in the *G. lucidum*. The abundance of this group of triterpenoids just follow ganoderic acids (16). To date, a total of 22 structurally distinct lucidenic acids have been identified in *G. lucidum*. Recently, Zheng et al. systematically reviewed the sources, contents, chemical structures, and pharmacological properties of lucidenic acids (16). In the current study, 13 lucidenic acids, including lucidenic acid A, B, C, D1, E2, F, G, J, K, L, M, and N, were identified. The majority exhibited significantly higher levels in WGL samples, including lucidenic acid A, D1, F, G, and J, as shown in Figures 8A–E. The combined content of all 13 lucidenic acids was notably greater in WGL samples, showing a 2.19-fold increase compared to SGL samples. This could be attributed to the differences in the growth substrates, environmental conditions, and other factors, such as microbial interactions inherent to the wood log cultivation method. These factors may have contributed to the synthesis and accumulation of these specific lucidenic acids. Further investigation into the metabolic pathways and gene expression related to these compounds in the two cultivation methods could provide deeper insights into the underlying mechanisms.

Ganoderic acids are a group of triterpenoid compounds with a pentacyclic triterpenoid backbone, characterized by various functional groups such as hydroxyls, ketones, and esters attached to the structure. These compounds exist in various structural forms, labeled as ganoderic acid A, B, C, etc. (32). They constitute a prominent and essential class of triterpenoids within *G. lucidum*. This group of triterpenoids are known for their diverse pharmacological properties, which include anti-inflammatory, antioxidant, immunomodulatory, and anti-tumor effects. These compounds have attracted substantial attention from researchers and have been the subject of numerous studies exploring their potential therapeutic applications. Liang et al. (12) have comprehensively reviewed the diverse ganoderic acids isolated and characterized in *G. lucidum*, as well as their associated health benefits. The diversity and abundance of triterpenoids can vary based on the cultivation methods employed, which in turn could influence the overall quality and potential benefits of *Ganoderma lucidum* products. This study identified nine specific ganoderic acids, including ganoderic acid A, alpha, C1, delta, DM, E, O, X, and xi across all analyzed samples. One of the key findings of this study is the differential abundance of specific ganoderic acids between the two cultivation methods. Ganoderic acid A and alpha were found to be more abundant in SGL samples, as illustrated in Figures 7C,D. This result suggests that the substitute cultivation method might favor the biosynthesis of these particular triterpenoids. Conversely, ganoderic acid E and O exhibited higher abundance in WGL samples, as shown in Figures 8F,G. This observation implies that the wood log cultivation method could influence the biosynthesis or accumulation of these triterpenoids. When considering all nine ganoderic acids, the total contents in WGL and SGL samples were comparable, slightly higher in WGL samples by 1.19-fold. The differences in nutrient availability and microenvironment within the cultivation bags during the cultivation process may contribute to the observed differences in ganoderic acid profiles.

In addition to ganoderic acids, lucidenic acids, ganosporelactones, and ganoderols, seven other triterpenoids, including polyporusterone F, ixocarpanolide, 11-anhydro-16-oxoalisol A, ganoderic acid O, 24-epi-brassinolide, pomolic acid, and verazine were also found to be in significant different abundance in the two types of samples (Table 1). The information of their presence in fruiting body of *G. lucidum* is relatively limited. More studies are required to further explore their contribution to the health benefits to *G. lucidum,* as well as the underlying reason for their differentiating abundance in SGL and WGL samples.

## 4 Conclusion

In this study, an untargeted metabolomic approach based on UHPLC-Q-Orbitrap-MS was employed to reveal the triterpenoid variation within the fruiting bodies of *G. lucidum* cultivated through wood log and substitute methods. A total of 96 terpenoid compounds were tentatively identified. As revealed by PCA and HCA, distinctive differences in triterpenoid profiles between these two types of samples were found. Additionally, we identified 17 differentiating triterpenoid compounds between them. The variations in the abundances of specific triterpenoids, such as lucidenic and ganoderic acids, between SGL and WGL samples highlight the potential of optimizing cultivation conditions to enhance the yield of specific bioactive compounds. Future studies are warranted to explore the genetic, biochemical, and environmental factors influencing triterpenoid synthesis in *G. lucidum*. Notably, this study also emphasizes that the cultivation method significantly impacts the triterpenoids composition in *G. lucidum*, influencing its antioxidant potency and potential therapeutic effects. The distinct triterpenoid profiles observed between WGL and SGL suggest diverse functional implications. Specifically, higher levels of lucidenic and ganoderic acids in WGL, known for anti-inflammatory and anti-tumor properties, suggest potential advantages in these health benefits. Conversely, SGL exhibiting elevated levels of ganosporelactone B and ganoderol A may suit specific therapeutic applications. Further investigation into triterpenoids and their effects could provide deeper insights into their respective functional use.

## Data availability statement

The original contributions presented in the study are included in the article/Supplementary material, further inquiries can be directed to the corresponding author.

## Author contributions

GL: Conceptualization, Funding acquisition, Investigation, Methodology, Project administration, Writing – original draft, Writing – review & editing. ZP: Conceptualization, Writing – review & editing. ZL: Methodology, Visualization, Writing – review & editing. WC: Data curation, Investigation, Methodology, Validation, Writing – review & editing. TY: Data curation, Investigation, Methodology, Validation, Writing – review & editing.
